# Sjögren’s syndrome: still not fully understood disease

**DOI:** 10.1007/s00296-014-3072-5

**Published:** 2014-07-02

**Authors:** Maria Maślińska, Małgorzata Przygodzka, Brygida Kwiatkowska, Katarzyna Sikorska-Siudek

**Affiliations:** Clinic of Early Arthritis, Institute of Rheumatology, Spartańska 1, 02-637 Warsaw, Poland

**Keywords:** Sjögren’s syndrome, Criteria, New therapies

## Abstract

Primary Sjögren's syndrome is an autoimmune disorder with external exocrine glands dysfunction and multiorgan involvement. The pathogenesis of primary Sjogren’s syndrome is still unclear; however, our knowledge of the involvement of different cells (e.g., B and T cells, macrophages and dendritic cells) and pathways (BAFF/APRIL and interferons) leading to the development of autoimmunity is continually expanding. For clinicians, the most frequent symptoms are dryness of eyes and mouth, but often the patients have musculoskeletal symptoms and systemic manifestations. However, the increased risk of lymphoproliferative disorders in this group of patients, most commonly B-cell marginal zone lymphoma, is particularly important. Recent separation of IgG4-related diseases and attempts to create further diagnostic criteria for pSS testify to the difficulties, and at the same time a large interest, in understanding the disease so as to allow the effective treatment. This article draws attention to the problems faced by the clinician wishing to securely identify pSS by using accurate laboratory biomarkers and useful imaging tools and predict the development of complications associated with this, still not fully understood, autoimmune disease.

## Introduction

It is known that primary Sjögren’s syndrome (pSS) is considered as an autoimmune disease based on limphocyte B hyper-reactivity and polyclonal production of immunoglobulins, and as a consequence, autoantibodies against pSS affected exocrine glands, especially lacrimal glands and salivary glands, but also other external glands such as the pancreas, mucous glands of the gastrointestinal and respiratory tract or bile secretion. In some patients, abnormal H+ secretion in the distal tubules has also been observed and caused distal renal tubular acidosis (type 1 RTA). In connection with the clinical symptoms which will result from the degree of attachment of these glands and epithelial injury, the pSS often uses the term “autoimmune epithelitis” or “autoimmune exocrinopathy” for better imaging of primary initiation sites of inflammation and autoimmunization.

## A brief history

First lacrimal and salivary glands enlargement was described in a lecture of a Polish surgeon Jan Mikulicz–Radecki in 1888. In 1925, French dermatologists Henri Gougerot described a few cases of atrophy of the salivary glands with dryness of eyes, mouth and vagina. Later in 1933, Swedish ophthalmologists Henrik Sjögren in his doctoral thesis described keratoconjunctivitis sicca, and his description became a basis for pSS picture.

### Epidemiology

It is estimated that primary Sjögren’s syndrome occurs from 0.1 to 3.0 % in general population. The disease is more common for women (female/male ratio 9:1), mainly between the ages of 40–60, with the disease most frequently occurring around 50 years of age.

**Fig. 1:**
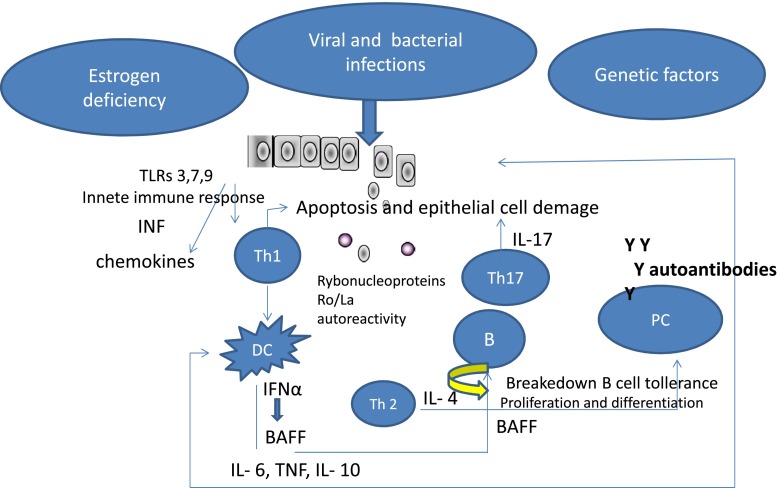
Sjögren’s syndrome outline of pathogenesis. *TLRs* toll-like receptors, *INF* interferon, *BAFF* B-cell activating factor, *IL* interleukin, *DC* dendritic cell, *B* B cell, *Th* T cell, *TNF* tumor necrosis factor, *PC* plasmocytic cell

*Pathogenesis* of pSS is not clear, nowadays several factors responsible for the development of the disease, such as genetic factors—genes implicated in B cell or B-cell activation factor (BAFF) known also as B-Lymphocyte Stimulator (BLyS), lymfotoxin α and β and TNF (tumor necrosis factor) are taken into account. Furthermore, it is presumed that genetic predisposition to increase in type I interferon (IFN) may explain the IFN signature and activation of type I IFN signaling in salivary gland and peripheral blood in pSS patients. The HLA-B8, of HLA-Dw3 and HLA-DR3 and DRw52 have also been reported in pSS patients [1, 2]. Another factor responsible for the development of pSS is an infection caused mainly by Epstain–Barr virus (EBV), human T-cell lymphotrophic virus type-1 (HTLV-1), Cytomegalovirus (CMV) and Hepatitis C virus (HCV). Also neurohormonal disturbances with sex hormones and its receptors dependent on hypothalamic–pituitary–adrenal axis (HPA or HTPA axis) interfere with the ratio of estrogens to androgens and affect steroid-dependent cells like epithelial cells and other cells involved in the immune response [3]. In pSS patients, lower basal secretion of ACTH and cortisol has been found. In addition, hypothalamic–pituitary–gonadal (HPG) axis by estrogen deficiency can be responsible for a local autoimmune exocrinopathy [4]. It is assumed that the infection as the trigger (most commonly viral) and other environmental factors caused the disorganisation of epithelial cells. First, due to innate immune response, virus infection is recognized by pattern recognition receptor (PRR) and activates toll-like receptors (TLR) pathway (e.g., TLR 3, 7, 9). After the activation, the innate immune response TLR, cell apoptosis and SS-A RNA complexes stimulate plasmocytic dendritic cells (pDCs) which produce high level of interferons (IFNs) and IFNs as strong stimulators of BAFF production by epithelial cells, monocytes and neutrophils dendritic cells leading to proliferation and differentiation of B cells and production of autoantibodies. It is suggested that glandular cell apoptosis triggered by viral infection (EBV, HCV and HIV) leads to progressive damage of glands and their dysfunction with reduced secretion and the appearance of classic clinical symptoms. Damaged epithelial cells release autoantigens, especially Ro/SSA and La/SSB, which create autoimmunity and autoantibodies secretions. The presence of Ro/SS-A antibodies (anti-Ro52 and anti-Ro60) is correlated with longer duration of pSS, bigger destruction of the glands and extraglandular manifestation. Plasmocytoid dendritic cells (pDCs) migrate into the site of damage. pDC is the source of type I INF and initiates the activation of B cell by B-cell activation factor (BAFF) pathway. However, epithelium is infiltrated mainly by CD 4+ limphocytes T subtype and immune response is balanced toward Th1 response and also Th17—with interleukin 17(IL-17) as a main cytokine. Th1 cells produce interferon gamma (IFN γ) which induces plasminogen activator system and together with IL-17 promotes local inflammation. In advanced stages of inflammation, B cells have been detected in salivary glands or other places in exocrine system. Recent study suggests that IL-7 from IL-7+ peripheral blood T cells may contribute to the stimulation of Th1 and Th17 cytokines [5]. It has been recently discovered that newly identified cytokine IL-34 promotes overexpression of CD 14+ monocytes in salivary glands. IL-34 and CSF-1 (colony stimulating factor-1) stimulate survival, proliferation and differentiation of monocytes, macrophages, dendritic cells, Langerhans cells and osteoclasts—cells which have the ability to phagocytose [6]. Studies have shown that also IL-21, cytokine produced by activated CD 4+ T limphocytes and NKT cells play a role in pathogenesis of pSS and correlate with lymphocytes infiltration of salivary gland as well as the presence of autoantibodies and level of gammaglobulins. This cytokine stimulates TH1 and Th17 differentiation and synergistically with BAFF stimulates B-cells differentiation [7]. As in many autoimmune diseases, the increased level of IL–6 has been found in pSS. IL-6 is one more cytokine which enhances lymphocytes B growth and maturation. As well as IL-6, it has a strong influence on persistence of inflammation [8]. The production of IL-6 by monocytes in pSS is strongly stimulated by INF γ. The increased activity of IL-6 may influence the clinical symptoms, and increased IL-6 has been reported in saliva, serum and tears [8] The break of T cells tolerance by viral infection leads to the activation of self-reactive T cells which by BAFF stimulate activation and maturation of B cells. Extended B-cell activation by BAFF generates self-reactive memory B cells which serve as an antigen-presenting cells to T cells. The crucial role of B limphocytes in pSS seems obvious—all pathways lead to powerful stimulation of B-cells maturation and to their hyper-reactivity, polyclonal production of gammaglobulins and the emergence of autoantibodies, which are B cell related markers for pSS. At first stage of inflammation, macrophages and T cells are infiltrating salivary glands, but over the time in the glands accumulate memory B cells and their level decreases in peripheral blood, while the level of naïve and transitional B cells increases. Interesting work presented by Bohnhorst et al. [10] showed disturbances in subset of B cells in pSS and differences in proportions between naïve B cells and memory cells in peripheral blood [9, 11]. Baldini and collegues [12] assessed a prevalence of vitamin D deficiency in early stage of the disease, considered the impact of hypovitaminosis D in clinical symptoms and disease activity, expressed by the symptoms of dryness and concluded that hypovitaminosis D is relatively frequent in patients with pSS. The investigators speculated that vitamin D may have the effect on Th1 cells and regulatory limphocytes, dendritic cells and CD 8+ T cells and plays a role in pSS pathogenesis. But the fact that vitamin D deficiency may be an independent and associated with reduced supply and reduced synthesis of this vitamin in relation to the living conditions and nutrition cannot be exclude. In another work, Agmon Levin et al. [13] showed the relationship between reduction of level of vitamin D and the occurrence of peripheral neuropathy and also of nonHodgkin lymphoma. The pathogenesis of Sjögren’s syndrome schematic is shown in Fig. 1

#### Autoantigens and autoantibodies

The most commonly present autoantibodies in pSS are antibodies against cellular ribonucleoprotein antigens Ro/anti-SS-A and LA/SS-B. These autoantibodies are not specific for pSS, only but their presence is associated with severe glandular manifestations, longer duration of the disease, presence of splenomegaly, lymphadenopathy, vasculitis and high infiltration of salivary glands. Ro52 antibodies were reported in about 80 % of pSS patients. In pSS, rheumatoid factor (RF) correlating with early stage of disease and onset of the disease at a younger age is often appearing. RF is present in about 74 % of patients with pSS and may also be associated with extraglandular symptoms such as arthritis. In 17 % of pSS anti-centromere antibodies (ACA), which are more specific for limited systemic sclerosis, were found. Their presence may signal an overlap syndrome [14].

The prevalence of antinuclear antibodies (ANAs) in pSS reaches 80 %, together with RF, in the case of the absence of anti-Ro/La along with histopathological confirmation or confirmation of ocular lesions allows make pSS diagnosis according to the ACR criteria. ANAs are also a predictor of internal organ involvement and the development of lymphoproliferative disorders. In pSS, M3 muscarinic acetylocholine receptor (M3R) is expressed in exocrine glands and its activation is important in the process of exocrine secretion. Sumida and colleagues found that M3R reactive T cells (Th1 and Th17) occur in 40 % in peripheral blood of patients with pSS and autoantibodies against M3R may play an important role in the development of autoimmune sialadenitis [15, 16]. In pSS patients, anti-mitochondrial antibodies (AMA), which are characteristic for primary biliary cirrhosis (PBC), can occur. In these patients, changes in the liver are more likely to appear, with periductal lymphocytic infiltrating analogously the changes in salivary glands. Abnormal liver function test (increased AST and/or ALT, AP and/or bilirubin) is common in pSS as extraglandular manifestation of the disease as well as overlapping with another disease as PBC or autoimmune hepatitis [16, 17]. Anti-smooth muscle antibodies (ASMA) are characteristic for autoimmune type 1 hepatitis which can also be found in pSS patients [18]. Also autoantibodies against carbonic anhydrase (anti-CAII) are present in pSS and are associated with distal renal tubular acidosis [19]. The anti-cyclic citrulinated peptide antibodies (anti-CCP), which are highly specific for RA, are not frequent in patient with pSS. The presence of anti-CCP antibodies may be associated with joint erosions and synovitis. Also it cannot be excluded that the patient who has anti-CCP antibodies and rheumatoid factor (RF-IgM) will develop symptoms of RA in the future [20]. In addition, autoantibodies with direct cytoskeletal proteins-α and β fodrin and proteasomes have been found in patients with pSS. The current studies showed additional autoantibodies in pSS to salivary gland as follows: protein 1 (SP-1), carbonic anhydrase 6 (CA6) and parotid secretory protein (PSP) [21]. Organ-specific antibodies, such as anti-thyroglobulin (anti-TG) and anti-thyroid peroxidase antibodies (anti-TPO), most characteristic for autoimmune thyroiditis, were also found. D’Arbonneau and colleagues reported that thyroid disease was more frequent in pSS patients in up to 30 % of patients compared with 4 % of the control group [22]. However, the presence of anti-TPO and TG antibodies in pSS may also not be related to the coexistence of Hashimoto’s disease [23].

#### Clinical picture of the disease

The clinical picture of pSS includes symptoms of dryness and extraglandular manifestations with organs involvement. Patients’ complaints of the dryness are the basis for suspicion. The dryness concerns first of all the mouth, eyes and vagina—the latter especially in postmenopausal women. The clinical signs are presented in Table 1. Among laboratory findings, cytopenias (leucopenia, anemia and low platelet count) are the most typical. Elevated erythrocyte sedimentation rate (ESR), hypergammaglobulinemia, ANAs, RF, anti-Ro/SS-A, anti-B/SSB, decreased level of complement component C4 and cryoglobulins are more often laboratory abnormalities in patients with pSS.

**Table 1:** Clinical features of primary Sjögren’s syndrome

*Dryness*	
Xerostomia (dry mouth)	Diminished secretion of saliva
	Troubles with swallowing
	Dental caries
	Fungal and bacterial infections
Xerophthalmia (dry eyes)	Persistent irritation keratoconjunctivitis sicca
	Destruction of cornea
	Decrease of vision
*Extraglandular manifestations*	
General symptoms	Fatigue
	Fever
	Weight loss
Musculoskeletal features	Myalgia
	Arthralgia
	Arthritis—nonerosive, also rheumatoid arthritis—like myositis
Respiratory tract	Cough—dryness of trachea or bronchitis
	Intestinal like disease
Gastrointestinal system	Dysphagia (dryness of pharynx and esophagus), gastrointestinal reflux
	Chronic gastritis with atrophy
	Liver involving (rather mild)
	Symptoms of PBC and AIH (autoimmune hepatitis)
	Celiac-like disease
Urinary tract	Distal renal tubular acidosis (RTA type 1)
	Nephrocalcinosis (in same cases due to RTA)
	Nepritis/glomerulonephritis
	Chronic renal insufficiency
Vessels and skin	Reynaud’s phenomenon (scleroderma like patterns with ACA antibodies)
	Vasculitis
	Urticaria, palpable purpura, annular erythrema
Neurological manifestations	Peripheral sensory or motor-sensory polyneuropathy,
	Cranial neuropathy,
	Mononeuritis multiplex
	Sensonarineural hearing loss
	SM-like syndrome
Psychiatric	Depression
	Anxiety
Cardiac	Pericarditis
	Pulmonary hypertension

### Classification criteria for pSS and tools useful in the diagnosis

Classification criteria of pSS have evolved from 1960s till new proposed in 2012. Since 2002, clinicians used criteria established and revised by American–European Consensus Group (AECG), which are based on six points: subjective and objectives features of dry eyes and dry mouth, the patient’s need to fulfill at least three subjective or two objective features and the presence of Ro/SS-A or La/SS-B antibodies or both, or inflammation in minor salivary glands with focus score (FS) equal or more than 1. FS is defined as 50 inflammatory cells in a 4-mm^2^ glandular section in material from minor salivary gland biopsy (SGB). Newly proposed ACR classification criteria for pSS assumed that in the cases with Ro/SS-A and La/SSB negative, but with symptoms suggestive for pSS and FS >1 in SGB the presence of ANAs titer above/equal to 1:320, and the presence of rheumatoid factor is sufficient to establish a diagnosis [24, 25]. This year Rasmussen et al. [26] compared new ACR criteria and AECG criteria and showed no significant differences and assumed ACR criteria sensitivity to 87.5 (95 % CI 82.9–90.9), specificity 93.4 (95 % CI 86.8–94.00, negative predictive value (NPV) 90.7, positive predictive value (PPV) 91. Among the 268 patients, who have met ACR criteria, 8.9 % did not fulfill AECG criteria, and among 279 patients, who fulfilled AECG criteria, 35 were not evaluated using ACR criteria. The differences are due to the fact that not all patients had keratoconjunctivitis sicca or positive serology and histopathological changes did not occur simultaneously. On the other hand, the patients that met only ACR criteria have been negative for Ro/La antibodies, but RF and ANAs positive. Comparative summary AECG criteria and ACR are shown in Table 2.

**Table 2:** Comparison of AECG and ACR proposed 2012 criteria for pSS [24–26]

The revised AECG criteria 2002	ACR proposed criteria 2012
Subjective symptoms	Subjective symptoms
1. Ocular symptoms Persistent dry eyes for more than 3 months? Recurrent sensation of sand or gravel in the eyes? Using tear substitutes more than three times a day?	Not included
2. Oral symptoms Feeling of dry mouth for more than 3 months? Recurrent or persistent swollen salivary gland? Frequent drinking to aid swallowing?	

Objective symptoms/examinations	Objectives symptoms
3. Schirmer’s test without anesthesia (<5 mm/5′ Rose Bengal score >4 in van Bijsterveld’s scoring system	1. Positive serum autoantibodies to Ro/SS-A or La/SS-B or both or positive rheumatoid factor and ANA ≥ 1:320
4. Histopathology-focus score ≥ 1	2. Salivary gland biopsy-focus score ≥1
5. Slivary gland involvement—positive results in one of: Unstimulated salivary flow (<1.5 ml in 15 min Sialography showing the presence of sialectasis without destructions of major ducts Scintigraphy with reduced uptake and concentration and/or excretion of tracer	3. Keratoconjunctivitis sicca with ocular staining score in individuals who is not using eye drops for glaucoma, and has not corneal surgery or cosmetic lid surgery in the last 5 years
6. Autoantibodies to Ro/SS-A or La/SS-B or both	

Exclusions	Exclusions
Head and neck radiation	Head and neck radiation
Hepatitis C infection	Hepatitis C infection
AIDS (acquired immune-deficiency syndrome)	Sarcoidosis
Pre-existing lymphoma	Amyloidosis
Sarcoidosis	AIDS (acquired immune-deficiency syndrome)
GVH (Graft versus Host) disease	GVH disease
Use of anticholinergic drugs	IgG4-related diseases

Diagnosis of pSS according to AECG criteria requires the assessment of salivary glands or salivary gland scintigraphy or sialography which is a specific, but invasive method of imaging. Unstimulated salivary flow and sialometry are other diagnostic tools to assess the function of salivary glands [27]. ACR criteria avoid such methods, limiting the estimation of salivary glands for histopathological examination. However, for clinicians, especially in the case of lack of patient’s consent for salivary gland biopsy, it is important to have a simple, available and noninvasive diagnostic tool for the assessment of salivary glands. It appears that salivary gland ultrasonography (SGUS) meets these assumptions. SGUS is relatively cheap, available and can be use not only for making a diagnosis, but also for controlling a disease progression and conversion into lymphoma. In Theander and Thomas study, 105 patients with pSS and 57 individuals in control group were evaluated by SGUS [28]. The authors used a simplified scoring system to evaluate salivary glands, taking into account the parenchymal echogenicity and inhomogeneity. This scoring system has been proposed by Hocevar [29] from normal echogenicity to next steps of hypoechoics lesions. For pSS, the score of 2 or 3 is characteristic. This method of scoring seems to be good for more advanced stages of the disease and can predict, together with laboratory findings, such as positive Ro/La antibodies, the presence of RF and ANAs in more severe disease and a higher risk of lymphoma development. Other researchers evaluate glands size, clearness of borders and vascularity, but it is currently believed that only parenchymal homogeneity is appropriate for the diagnosis of changes in pSS [30]. Other methods, such as MR sialography and computed tomography (CT), may have limited availability and are undoubtedly more expensive. The basis of the diagnosis and a strong argument of inflammation and lymphocytes infiltration of salivary glands is histopathological examination. Standardization of techniques of minor salivary glands biopsy makes it possible to reduce the risk of complications and even increase the value of this study. Varela-Centelles et al. [30] suggested the use of S forceps for better positioning of the lower lip than chalazion forceps and to avoid postoperative complications [31]. In histopathological assessment in pSS focal infiltrates, CD 4+ T cells in early stage and more memory B cells in next stages are observed, further aggregation of limphocytes, organized lymphoid follicle, ectopic germinal center-like structure (GC) and in some cases lymphoma picture. The degree of organization of infiltration correlates with the severity of the disease, large activity of cytokines in situ and by the presence of autoantibodies [32].

#### Sjögren’s syndrome and IgG4-related diseases

The cases of lacrimal and salivary glands being affected by the disease, that manifests itself with the symptoms of dryness and is accompanied by autoimmune pancreatitis and renal disease (IgG4-related kidney disease), have for years been recognized as a subtype of pSS. Japanese clinicians and scientist first demonstrated that such patients show lymphoplasmocytic infiltrations with IgG4 plasma cells. Additionally, diffuse or local organ enlargement, fibrosis and/or hyperplastic lesions with symptoms of organ damage have been observed in this group. The term “IgG4-related diseases” was finally proposed with classification criteria for IgG4 RD established. IgG4 RD affects mainly elderly man (>60 years old) [33]. The serum level of IgG4 is significantly (>135 mg/dl) increased, and a tissue biopsy reveals infiltrations of IgG4 plasma cells, with IgG4/IgG ratio exceeding 40 %. Patients’ plasma tests seronegative for the presence antinuclear antibodies, including SS-A and SS-B. Hypocomplementemia, high serum levels of polyclonal gammaglobulins with increased level of IgG4 and IgE gammaglobulins may also occur. The histopathological assessment shows plasmocyte and lymphocyte infiltrations and may reveal fibrosis. Establishing the diagnosis of IgG4 RD requires first of all the exclusion of malignancy, for which task histopathological examination is vital. Other diseases, chiefly pSS, but also Castelman’s disease, ANCA-associated vasculitis, idiopathic retroperitoneal fibrosis, sarcoidosis should also be considered as an alternative diagnosis and excluded, using diagnostic criteria for each of the listed diseases. Differences between pSS and IgG4 RD are presented in a Table 3.

**Table 3:** Differences between pSS and IgG4 RD

Primary Sjogren’s syndrome	IgG4-related diseases
Age between 30 and 50	Age >60
Mainly women	Mainly men
Salivary glands enlargement may occur	Organ enlargement (local or diffuse)
Symptoms of dryness	Symptoms of dryness may occur
Presence of ANA antibodies	Absence of antinuclear antibodies
Presence of anti-SS-A, SS-B antibodies	Absence of anti-SS-A SS-B antibodies
Polyclonal hypergammaglobulinemia	Polyclonal hypergammaglobulinemia
Normal level of IgG4	Significantly increased level of IgG4
Mainly CD 4+ T lymphocyte infiltrations	Mainly IgG4-positive plasmocytic infiltrations IgG4/IgG ratio >40 %
Treatment: symptomatic and systemic (antimalarics, methotrexate, corticosteroids, cyclosporine A, azathioprine, cyclophosphamide)	Treatment: steroids (no response to steroid treatment indicates incorrect diagnosis)

#### HCV infection and primary Sjögren’s syndrome: a topic for discussion

Patients infected with HCV may have dryness and cryoglobulinemia, but Ro/SS-A and La/SS-B antibodies are rarely present in the HCV infection. The discussion invokes the potential role of HCV infection in the pathogenesis of pSS. Various studies depict the presence of HCV in 14–19 % of patients with pSS. Ramos-Casals et al. [34] conducted a study of 137 patients diagnosed with pSS and co-infected with HCV. In 50 % of this group, cryoglobulins were present, and only 23 % had antibodies to Ro/SS-A and/or La/SS-B, 65 % had ANA antibodies. The authors suggest that chronic HCV infection must be considered as a criterion for exclusion pSS mainly by influencing the development and course of pSS and also proposed the term secondary to HCV Sjögren’s syndrome. Also at work, Madaliński et al. [35] who examined 104 patients diagnosed with pSS searched for markers of infection HCV and in 19.2 %—HCV antibodies were found and 4.8 % patients had genetic material of the virus (HCV-RNA); researchers also suggested distinguish disease as different from pSS.

#### Risk of lymphoma and development of other cancers

Patients with pSS have a significantly higher risk of the development of EBV-associated lymphomas. EBV DNA that was found in saliva and B cells. This presence of epithelial cells in salivary glands suggests active infection (reactivation of EBV). The development of lymphoma has been described in 4–7 % of patients with pSS [36, 37]. This applies particularly to the development of MALT (mucosa-associated lymphoid tissue) lymphomas and also splenic marginal zone lymphomas (SMZL) as well as nodal marginal zone lymphoma (NMZL) [37]. For years, a risk factors associated with the development of lymphomas have been analyzed—taking into account both clinical symptoms and potential biomarkers associated with the development of lymphoproliferation, which are presented in the Table 4 [38, 39].

**Table 4:** Clinical manifestations and biomarkers associated with lymphoma development in pSS

Clinical manifestations	Potential biomarkers
Vasculitis	Cryoglobulins (mixt cryoglobulinemia)
Salivary gland enlargement	Low C4 complement component
Salivary gland/parotid swelling	Anti-Ro/SS-A antibodies
Lymphadenopathy	Leukopenia
Splenomegaly	Presence of RF expressing B cells
Peripheral neuropathy	*Flt3-L (Fms-like tyrosine kinase3 ligand)
Long duration of pSS	Higher levels of BAFF/BLyS
Histopathology: germinal-like structures in minor salivary glands biopsy	

*Fms-like tyrosine kinase ligand is a cytokine-stimulating growth of bone marrow and blood progenitor cells, and Flt 3 expression is described in lymphoma cells [40].

#### Treatment of pSS

*Symptomatic treatment* In the case of symptoms of dry eyes, artificial tears are used, topical drops with cyclosporin A and corticosteroids. In more severe cases, it is necessary to close the lacrimal ducts. Dry mouth treatment is limited to recommending drinking larger quantities of fluids, gentle acidification of fluids and the use of artificial saliva. Systemic administration of drugs to enhance salivating includes agonists muscarinic receptors: pilocarpine and cevimeline [41]. Sometimes *N*-acetylocysteine may be useful. It is important to treat fungal infections of the oral cavity. Vaginal dryness and dysperunia should be treated with lubricants, propionic acid gels, and sometimes, in the case of menopause, vaginal cream with estrogens.

*Systemic treatment* is based on antimalaric drugs such as hydroxychlorochine and especially in the case of arthritis methotrexate, corticosteroids, cyclosporine A and azathioprine. In severe cases, with life threat involving organs (renal involvement, neurological, pulmonary involvement and vasculitis) cyclophosphamide, infusions of immunoglobulin, plasmapheresis is used. Patients with renal tubular acidosis should receive sodium and/or potassium.

*Potential new therapies* In view of the demonstrated key role of B cells—their hyperstimulation and overproduction of autoantibodies in pSS—B cells have become a logical target for therapy. Rituximab (RTX), chimeric antibody against anti-CD20, has proven effective in reducing both the destruction of the salivary glands, reducing inflammatory infiltration in salivary glands, which not only shows the effect on the depletion of B cells, but also on T lymphocytes. Also after infusion of rituximab, significant decrease of rheumatoid factor Ig-M in serum was observed [42]. RTX treatment had also an influence on reduction of symptoms of dryness, fatigue and artralgias. Efficacy of B-cell depletion in pSS initiated further search for drugs-affecting B cells and BAFF/BlyS as a main stimulator of B cells has become a new target of therapy. Belimumab is a monoclonal antibody which inhibits the biological activity of B-Lymphocyte stimulator (BLyS), currently accepted in SLE treatment. BELLIS open label phase II study showed benefits of belimumab in patients with pSS, but further studies are needed **[** 43, 44]. Epratuzumab is the humanized anti-CD22 monoclonal antibody that causes moderate B-cell depletion, therefore, can be another toll for pSS treatment. However, previous research showed clinical improvement with no influence on level of autoantibodies [45, 46]. The future of pSS treatment may be an IFN-related therapies and influence on IFN-related pathways. Mesenchymal stem cells (MSCs) transplantation, thanks to low immunogenicity and ability of multipotent stem cells to differentiate into various cells, seems to be promising method in pSS. The effectiveness of such treatment has been demonstrated in SLE and systemic sclerosis [47].

## Conclusions

Particular challenge is to make the pSS diagnosis in the early stages of the disease. The presence of typical pSS serological markers such as anti-SS-A and anti-SS-B antibodies at an early stage cannot be confirmed, which is taken into account in the newly proposed ACR classification criteria. Discoveries of novel cellular subsets and cytokines involved in pSS pathogenesis may become a new target for future therapies. Both gene transfer and MSC transplantation can find wider application in the pSS treatment.

## References

[1] Cruz-Tapias P, Rojas-Villarraga A, Maier-Moore S, Anaya JM (2012). HLA and Sjögren’s syndrome susceptibility. A meta-analysis of worldwide studies. Autoimmun Rev.

[2] Peri Y, Agmon-Levin N, Theodor E, Shoenfeld Y (2012). Sjögren’s syndrome, the old and the new. Best Pract Clin Rheum.

[3] Johnson EO, Skopouli FN, Moutsopoulos HM (2000). Neuroendocrine manifestations in Sjögren’s syndrome. Rheum Dis Clin North Am.

[4] Tzioufas AG, Tsonis J, Moutsopoulos HM (2008). Neuroendocrine dysfunction in Sjogren’s syndrome. NeuroImmunoModulation.

[5] Bikker A, Moret FM, Kruize AA, Bijlsma JW, Lafeber FP, van Roon (2002). IL-7 drives Th1 and Th17 cytokine production in patients with primary SS despite an increase in CD4 T cells lacking the IL-7Rα. Rheumatology (Oxford).

[6] Ciccia F, Alessandro R, Rodolico V (2013). IL-34 is overexpressed in the inflamed salivary glands of patients with Sjogren’s syndrome and is associated with the local expansion of pro-inflammatory CD14(bright)CD16+ monocytes. JA Rheumatology (Oxford).

[7] Kang KY, Kim HO, Kwok SK, Ju JH (2011). Impact of interleukin-21 in the pathogenesis of primary Sjögren’s syndrome: increased serum levels of interleukin-21 and its expression in the labial salivary glands. Arthritis Res Ther.

[8] Yoshimoto K, Tanaka M, Kojima M, Setoyama Y, Kameda H, Suzuki K, Tsuzaka K, Ogawa Y, Tsubota K, Abe T, Takeuchi T (2011). Regulatory mechanisms for the production of BAFF and IL-6 are impaired in monocytes of patients of primary Sjögren’s syndrome. Arthritis Res Ther.

[9] Cornec D, Saraux A, Pers JO (2014). Diagnostic occuracy of blood B-cell subset profiling and autoimmunity markers in Sjogren’s syndrome. Arthritis Res Ther.

[10] Bohnhorst JØ, Bjørgan MB, Thoen JE, Natvig JB, Thompson KM (2001). Bm1–Bm5 classification of peripheral blood B cells reveals circulating germinal center founder cells in healthy individuals and disturbance in the B cell subpopulations in patients with primary Sjögren’s syndrome. Immunology.

[11] Youinou P, Pers JO (2011). Disturbance of cytokine networks in Sjögren’s syndrome. Arthritis Res Ther.

[12] Baldini C, Delle Sedie A, Luciano N, Pepe P, Ferro F, Talarico R, Tani C, Mosca M (2013) Vitamin D in “early” primary Sjögren’s syndrome: does it play a role in influencing disease phenotypes? Rheumatol Int. doi:10.1007/s00296-013-2872-3 10.1007/s00296-013-2872-3 24097207

[13] Agmon-Levin N, Kivity S, Tzioufas AG (2012). Low levels of vitamin-D are associated with neuropathy and lymphoma among patients with Sjögren’s syndrome. J Autoimmun.

[14] Kyriakidis NC, Kapsogeorgou EK, Tzioufas AG (2003) A comprehensive review of autoantibodies in primary Sjogren’s syndrome: clinical phenotypes and regulatory mechanisms. J Autoimmun 51C:67–74 10.1016/j.jaut.2013.11.001 24333103

[15] Sumida T, Tsuboi H, Iizuka M, Hirota T, Asashima H, Matsumoto I (2014) The role of M3 muscarinic acetylcholine receptor reactive T cells in Sjögren’s syndrome: a critical review. J Autoimmun (4). doi:10.1016/j.jaut.2013.12.012 10.1016/j.jaut.2013.12.012 24397962

[16] Sumida T, Lizuka M, Asashima H, Tsuboi H, Matsumoto I (2012). Pathogenic role of anti-M3 muscarinic acetylcholine receptor immune response in Sjögren’s syndrome. Presse Med.

[17] Kaplan MJ, Ike RW (2002). The liver is a common non-exocrine target in primary Sjögren’s syndrome: a retrospective review. BMC Gastroenterol.

[18] Karp JK, Akpek EK, Anders RA (2010) Autoimmune hepatitis in patients with primary Sjögren’s syndrome: a series of two-hundred and two patients. Int J Clin Exp Pathol 25;3(6):582–6 PMC2907119 20661405

[19] Ono M, Ono M, Watanabe K, Miyashita Y (1999). A study of anti-carbonic anhydrase II antibodies in rheumatic autoimmune diseases. J Dermatol Sci.

[20] Atzeni F, Sarzi-Puttini P, Lama N (2008). Anti-cyclic citrullinated peptide antibodies in primary Sjogren syndrome may be associated with non-erosive synovitis. Arthritis Res Ther.

[21] Shen L, Suresh L, Lindemann M (2012). Novel autoantibodies in Sjogren’s syndrome. Clin Immunol.

[22] D’Arbonneau F, Ansart S, Le Berre R, Dueymes M, Youinou P, Pennec YL (2003). Thyroid dysfunction in primary Sjögren’s syndrome: a long-term followup study. Arthritis Rheum.

[23] Tunc R, Gonen M, Acbay O, Hamuryudan V, Yazici H (2004). Autoimmune thyroiditis and anti-thyroid antibodies in primary Sjögren’s syndrome: a case–control study. Ann Rheum Dis.

[24] Shiboski SC, Shiboski CH, Criswell L et al (2012) American College of Rheumatology classification criteria for Sjögren’s syndrome: a data-driven, expert consensus approach in the Sjögren’s International Collaborative Clinical Alliance cohort. Arthritis Care Res (Hoboken) A;64(4):475–87 10.1002/acr.21591 PMC3349440 22563590

[25] Vitali C, Bootsma H, Bowman SJ (2013). Classification criteria for Sjögren’s syndrome: we actually need to definitively resolve the long debate on the issue. Ann Rheum Dis.

[26] Rasmussen A, Ice JA, Li H et al (2014) Comparison of the American-European Consensus Group Sjögren’s syndrome classification criteria to newly proposed American College of Rheumatology criteria in a large, carefully characterized sicca cohort. Ann Rheum Dis 73(1). doi:10.1136/annrheumdis-2013-203845 10.1136/annrheumdis-2013-203845 PMC3855629 23968620

[27] Speight PM, Kaul A, Melsom RD (1992). Measurement of whole unstimulated salivary flow in the diagnosis of Sjögren’s syndrome. Ann Rheum Dis.

[28] Theander E, Mandl T. (2013) Primary sjogrens syndrome : the diagnostic and prognostic value of salivary gland ultrasonography using simplified scoring system. Arthritis Care Res (Hoboken) 10. doi:10.1002/acr.22264 10.1002/acr.22264 24339361

[29] Hocevar A, Amrozic A, Rozman B, Kveder T, Tomsic M (2005). Ultrasonographic changes of major salivary glands in primary Sjogren’s syndrome. Diagnostic value of the novel scoring system. Rheumatology (Oxford).

[30] Varela-Centelles P, Seoane-Romero JM, Sánchez-Sánchez M, González-Mosquera A, Diz-Dios P, Seoane J (2014) Minor salivary gland biopsy in Sjögren’s syndrome: a review and introduction of a new tool to ease the procedure. Med Oral Patol Oral Cir Bucal 1;19(1):e20–e23 10.4317/medoral.19131 PMC3909427 23986014

[31] Lida Santiago M, Seisdedos MR, García Salinas RN (2012). Frequency of complications and usefulness of the minor salivary gland biopsy. Reumatol Clin.

[32] Theander E, Vasaitis L, Baecklund E (2011). Lymphoid organisation in labial salivary gland biopsies is a possible predictor for the development of malignant lymphoma in primary Sjögren’s syndrome. Ann Rheum Dis.

[33] Okazaki K, Umehara H (2012) Are classification criteria for IgG4-RD now possible? The concept of IgG4 related diseases and proposal of comprehensive diagnostic criteria in Japan. Int J Rheum. doi:10.1155/2012/357971 10.1155/2012/357071 PMC3368488 22690221

[34] Ramos-Casals M, Loustaud-Ratti V, De Vita S, Zeher M, Bosch JA, Toussirot E, Medina F, Rosas J, Anaya JM, Font J, SS-HCV Study Group (2005). Sjögren syndrome associated with hepatitis C virus: a multicenter analysis of 137 cases. Medicine (Baltimore).

[35] Madaliński K, Godzik P, Zimmermann-Górska I (2009). Anti-HCV and HCV-RNA in patients with primary Sjogren’s syndrome. Przegl Epidemiol.

[36] Voulgarelis M, Dafni UG, Isenberg DA, Moutsopoulos HM (1999). Malignant lymphoma in primary Sjögren’s syndrome: a multicenter, retrospective, clinical study by the European concerted action on Sjögren’s syndrome. Arthritis Rheum.

[37] Baimpa E, Dahabreh IJ, Voulgarelis M, Moutsopoulos HM (2009). Hematologic manifestations and predictors of lymphoma development in primary Sjögren syndrome: clinical and pathophysiologic aspects. Medicine (Baltimore).

[38] Thiebelmont C, Bertoni F, Copie-Bergman C, Ferreri AJM, Ponzoni M (2013). Chronic inflammation and extra-nodal-zone lymphomas of MALT-type. Semin Cancer Biol.

[39] Quartuccio L, Isola M, Baldini Ch et al (2013) Biomarkers of lymphoma in Sjogren’s syndrome and evaluation of the lymphoma risk in prelymphomatous conditions: results of a multicenter study. J Autoimmun 1–6. doi:10.1016/j.jaut.2013.10.002 10.1016/j.jaut.2013.10.002 24231556

[40] Baecklund E, Smedbyb KE, Suttonc LA, Askling J, Rosenquist R (2014). Lymphoma development in patients with autoimmune and inflammatory disorders—what are the driving forces?. Semin Cancer Biol.

[41] Barclay L (2010). Treatment of primary Sjogren’s syndrome reviewed. JAMA.

[42] Meijer JM, Meiners PM, Vissink A (2010). Effectivnes of Rituximab treatment in primary Sjögren’s syndrome. Arthritis Rheum.

[43] Mariette X, Seror R, Quartuccio L et al (2013) Efficacy and safety of belimumab in primary Sjögren’s syndrome: results of BELLIS open label phase II study. Ann Rheum Dis 1–6. doi:10.1136/annrheumdis-2013-203991 10.1136/annrheumdis-2013-203991 24347569

[44] Levasque MC (2009). Translational mini-review series on B cell-directed the rapies: recent advances in B cell-directed biological therapies for autoimmune disorders. Clin Exp Immun.

[45] Steinfeld SD, Tant L, Burmester GR (2006). Epratuzumab (humanised anti-CD22 antibody) in primary Sjögren’s syndrome: an open-label phase I/II study. Arthritis Res Ther.

[46] Vossenkämper A, Lutalo PM, Spencer J (2012). Translational Mini-Review Series on B cell subsets in disease. Transitional B cells in systemic lupus erythematosus and Sjögren’s syndrome: clinical implications and effects of B cell-targeted therapies. Clin Exp Immunol.

[47] Xu J, Wang D, Liu D, Fan Z et al (2012) Allogeneic mesenchymal stem cell treatment alleviates experimental and clinical Sjögren syndrome. Blood 11;120(15):3142–51 10.1182/blood-2011-11-391144 PMC3471521 22927248

